# The prognostic value of lactate dehydrogenase levels in colorectal cancer: a meta-analysis

**DOI:** 10.1186/s12885-016-2276-3

**Published:** 2016-03-25

**Authors:** Guanghua Li, Zhao Wang, Jianbo Xu, Hui Wu, Shirong Cai, Yulong He

**Affiliations:** Department of Gastrointestinal Surgery, First Affiliated Hospital of Sun Yat-sen University, 510080 Guangzhou, Guangdong Province People’s Republic of China

**Keywords:** Lactate dehydrogenase, Colorectal cancer, Prognosis, Meta-analysis

## Abstract

**Background:**

The prognostic value of lactate dehydrogenase levels in the prognosis of colorectal cancer patients has been assessed for years, although the results remain controversial and heterogeneous. Thus, we comprehensively reviewed the evidence from studies that evaluated lactate dehydrogenase levels in colorectal cancer patients to determine their effect.

**Methods:**

The following databases were searched in September 2014 to identify studies that evaluated the prognostic value of lactate dehydrogenase levels in colorectal cancer: PubMed, EMBASE, and the Cochrane Central Register of Controlled Trials. We extracted hazard ratios (HRs) and the associated 95 % confidence intervals (CIs) from the identified studies, and performed random-effects model meta-analyses on the overall survival (OS) and progression-free survival (PFS). Thirty-two studies with a cumulative sample size of 8,261 patients were included in our analysis.

**Results:**

Our meta-analyses revealed that high levels of lactate dehydrogenase were associated with poor OS (HR, 1.75; 95 % CI, 1.52–2.02) in colorectal cancer patients. However, this effect was not obvious in the OS of non-metastatic colorectal cancer patients (HR, 1.21; 95 % CI, 0.79–1.86). The prognostic value of lactate dehydrogenase levels on PFS was also not confirmed (HR, 1.36; 95 % CI, 0.98–1.87). Subgroup analyses revealed that the prognostic significance of lactate dehydrogenase was independent of study location, patient age, number of patients, metastasis, chemotherapy with anti-angiogenesis drugs, study type, or risk of bias.

**Conclusions:**

Our results indicate that high lactate dehydrogenase levels are associated with poor OS among colorectal cancer patients, although these levels are not significant predictors of PFS.

**Electronic supplementary material:**

The online version of this article (doi:10.1186/s12885-016-2276-3) contains supplementary material, which is available to authorized users.

## Background

Colorectal cancer (CRC) represents the third most common malignancy throughout the world [1]. The prognosis for late stage CRC is extremely poor, and survival is often measured in months once metastases are present. Moreover, despite the fact that advances in modern systemic therapies for CRC have resulted in improved survival, the failure rate in the adjuvant setting is 30 % for high risk Stage II and Stage III patients, and the overall response rate is only 60 % for patients with Stage IV CRC [2, 3]. Therefore, it is necessary to discover biomarkers that can identify patients that are at-risk for disease recurrence and survival.

Cancer cells rely heavily on aerobic glycolysis to support their growth, a process that is known as the Warburg effect [4, 5]. Lactate dehydrogenase plays an important role in this process by mediating the conversion of pyruvate and lactate, and this enzyme is an emerging anticancer target [6]. In addition, elevated lactate dehydrogenase levels are consistently reported as a prognostic factor for poor survival among several cancer groups [7]. The authors conducted a prospective study, including various cancer types (liver, lung, bone, brain etc.), symptoms, signs and other serological variables, to evaluate LDH’s value as a predictor of survival time in terminal cancer patients. Their results demonstrated that serum LDH level was significantly associated with survival time (HR = 2.087, *P* = 0.002) in patients with terminal cancer [7]. Although a large number of studies have been performed among patients with CRC, the prognostic value of lactate dehydrogenase levels among CRC patients remains controversial. Thus, we conducted this meta-analysis to evaluate the prognostic value of lactate dehydrogenase levels among CRC patients.

## Methods

### Search strategy and selection criteria

The following databases were searched in September 2014: PubMed, EMBASE, and the Cochrane Central Register of Controlled Trials. In addition, we examined the reference lists of relevant articles and review articles. No language restrictions or time limits were applied to the initial search. Search strategies, databases, and date ranges are provided in the supplemental material (Additional file 1). Eligibility criteria for inclusion in this meta-analysis were: [1] the study evaluated the correlation between lactate dehydrogenase levels and survival among CRC patients, [2] the study provided sufficient information for the estimation of hazard ratios (HRs) and their 95 % confidence intervals (CIs), and [3] the study was published in English, German, or French. Two reviewers (L.G.H. and W.Z.) independently screened the identified abstracts for eligibility, and disagreements were resolved by discussion. When multiple publications reported identical or overlapping patient cohorts (e.g., same authors, institutions), only the most informative study was included in the analysis.

### Data extraction

Two investigators (L.G.H. and W.Z.) independently extracted the following data from the eligible articles: first author, year of publication, study location, sample size, patient age, site of disease, stage of disease, Lactate dehydrogenase cut-off value, use of adjuvant chemotherapy, prognostic outcomes, use of multivariate models, and study type.

### Study quality assessment

The quality of the included studies was assessed using the modified risk of bias tool that is recommended by the Cochrane Collaboration, as previously described [8, 9]. Briefly, the criteria in Additional file 2 were used to assess the risk of bias of included studies. Each question is answered with “Yes” (indicating low risk of bias), “No” (indicating high risk of bias), and “Unclear” (indicating unclear or unknown risk of bias). The summary assessment of the risk of bias for the individual studies was carried out as follows: 1. Low risk of bias: Low risk of bias for all domains. 2.Unclear risk of bias: Unclear risk of bias for one or more domains. 3.High risk of bias: High risk of bias for one or more domains.

### Statistical analyses

The prognostic value of lactate dehydrogenase levels for survival was measured using HRs. If an HR and the associated standard error or CI was not reported, we approximated the HR using the statistical data that was provided in the article (e.g., individual patient data or survival plots) [10, 11]. The extracted HRs were pooled using a fixed-effects model (weighted with inverse variance) or a random-effects model [12]. Our method consisted of using the fixed-effects model with an assumption of homogeneity in the individual HRs. Heterogeneity between studies was assessed using the *χ*^2^ and I^2^ statistics. If the assumption of homogeneity was rejected, the random-effects model was used [13].

HR >1 indicated a worsened prognosis in the high lactate dehydrogenase group, and a minimum of 3 studies was required to perform the meta-analyses. Sensitivity analysis was also conducted using sequential omission of individual studies to evaluate the stability of the results. Funnel plot analyses were used to evaluate publication bias [14]. All analyses were performed using STATA version 10.0, and a *p*-value <0.05 was considered statistically significant.

## Results

### Baseline study characteristics

**Fig. 1:**
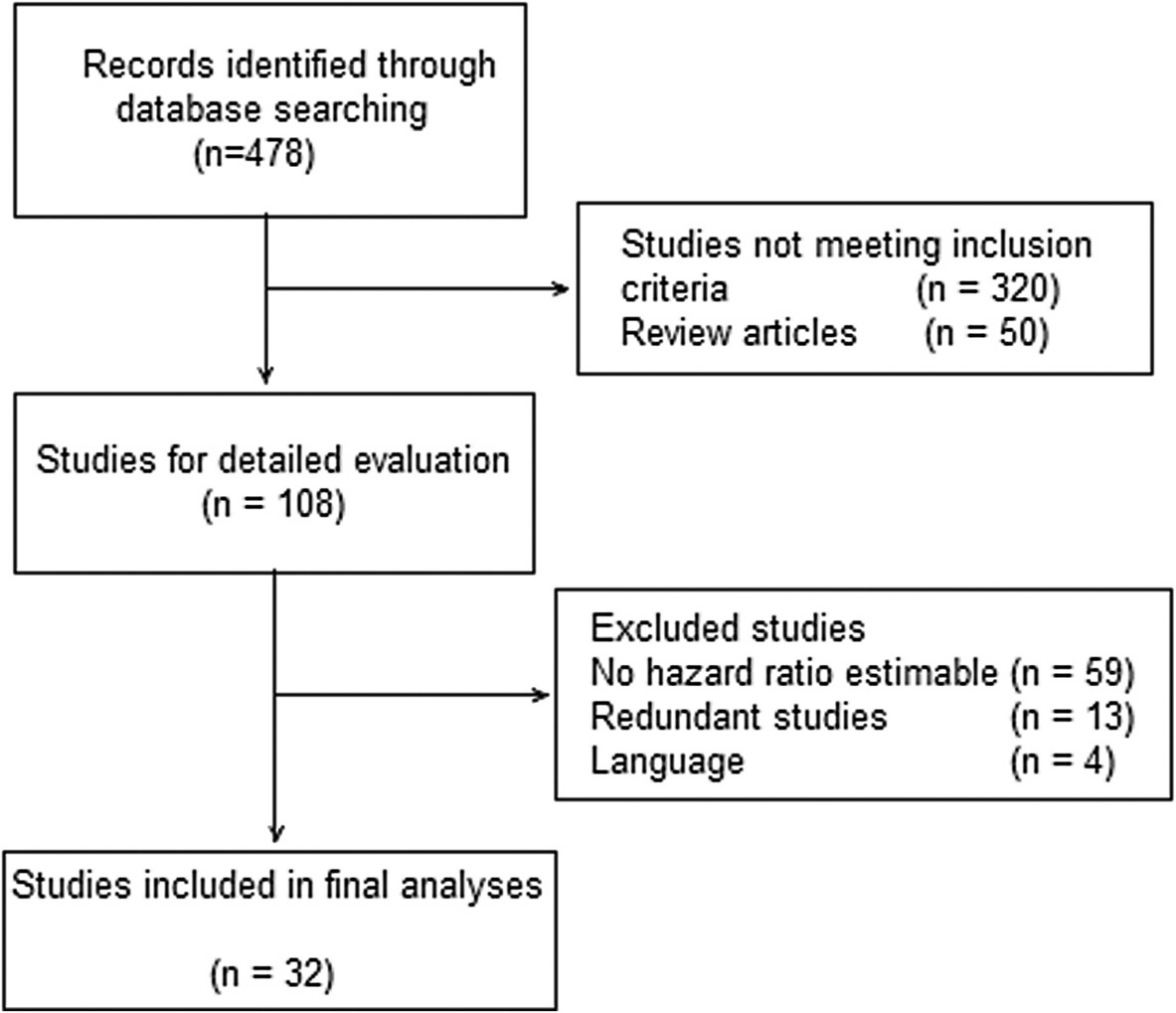
Flow chart for the study selection

We identified 32 eligible studies with a cumulative sample size of 8,261 patients (Fig. 1) [15–47]. The median study sample size was 157 patients (range, 31–855 patients), and all eligible studies were published between 1988 and 2014 (Table 1). Thirteen studies were excluded owing to the inclusion of a patient cohort that was also used in the other selected studies (studies that were excluded and included were [24, 48–59]). The extracted variables from the included studies are summarized in Table 1 (Abbreviations: FOLFOX, infusional fluorouracil, leucovorin, and oxaliplatin; FU, fluorouracil; IHC, immunohistochemistry; RCT, randomized controlled trial; NR, not reported; RMCS, retrospective multicenter cohort study; PSCS, prospective single-center cohort study; RSCS, retrospective single-center cohort study).

**Table 1:** Baseline characteristics of included studies

			Sample size			Age			LDH				
First author	Year	Country	Total	Colon	Rectum	Median	Range	Tumor stage	Cutoff	Detection method	Adjuvant chemotherapy	Suvival analysis	Outcome report
Agrawal	2013	USA	146	NR	NR	NR	<=50	IV	200U/L	serum	NR	Univariate	OS
Alonso-Espinaco	2014	Spanish	157	NR	NR	NR	28–82	mCRC	NR	serum	FOLFOX/XELOX	Univariate Multivariate	OS PFS
Asmis	2011	Canada	544	NR	NR	NR	NR	NR	NR	serum	Cetuximab-based	Univariate Multivariate	OS
Caputo	2014	Italy	96	88	6	NR	18–80	T2T3T4/M0	248U/L	serum	NO	Univariate	OS PFS
Cetin	2012	Turkey	168	NR	NR	NR	NR	mCRC	NR	serum	anti-VEGF therapy	Multivariate	OS
Chibaudel	2011	France	535	349	177	65	29–80	mCRC	NR	serum	Oxaliplatin-Based or Irinotecan-Based First-Line Chemotherapy	Univariate Multivariate	OS
Diouf	2014	France	620	398	211	NR	18–80	mCRC	NR	serum	FOLFOX4 or FOLFOX7	Univariate Multivariate	OS
Formica	2013	Italy	31	26	5	69	41–83	mCRC	245U/L	serum	FOLFORIN + bevacizumab	Multivariate	PFS
Galizia	2008	Italy	65	53	12	NR	28–84	IV with liver metastasis	450U/L	serum	fluorouracil, folinic and acid, and oxaliplatin/irinotecan	Multivariate	OS
Giessen	2013	German	215	136	79	61.8	32–78	mCRC/liver metastas	250U/L	serum	FUFURI or mIROX	Multivariate	OS
Giessen	2014	Italy	249	0	249	64.6	30.6–90.7	I-III	171	serum	Chemotherapy/Radiotherapy/Concomitant chemoradiotherapy	Univariate	OS
Hannisdal	1994	Norway	100	0	100	69	33–87	Local regional relapse ± metastasis	500	serum	chemoradiotherapy	Multivariate	OS
He	2013	China	239	171	68	57	18–83	mCRC	245U/L	serum	Folfox/Xelox/Folfiri/Xeliri	Multivariate	OS
Koukourakis	2006	UK	128	78	50	67	41–88	Dukes B,C,D	NR	IHC	NO	Univariate	OS
Koukourakis	2011	Greece	179	NR	NR	NR	28–83	mCRC	NR	serum IHC	FOLFOX4 + vatalanib/placebo	Univariate Multivariate	OS
Lin	2006	USA	66	NR	NR	62	30–86	mCRC	618	serum	XCEL ± Radiation	Univariate	OS
Lin	2005	China	45	34	11	32	18–39	Dukes B,C,D	230	serum	5-FU based chemotherapy	Multivariate	OS
Machida	2008	Japan	103	66	37	62	29–80	mCRC	300	serum	LV-modulated 5-FU/irinotecan + 5-FU	Univariate	OS
Maurel	2007	Spain	120	NR	NR	66	33–82	mCRC	450	serum	5-FU + oxaliplatin/irinotecan	Multivariate	OS
Mekenkam	2012	Netherland	803	538	260	63	27–84	Advanced stage (curative surgery)	NR	serum	capecitabine, irinotecan, oxaliplatin: Sequential VS Combination	Multivariate	OS

Among the 32 studies that used serum lactate dehydrogenase levels to investigate their influence on patient prognosis, 2 studies [29, 30] used an immunohistochemistry method, and 1 study [30] used serum levels and immunohistochemistry methods. Twelve studies were graded with a low risk of bias (Additional file 2). Our analysis of lactate dehydrogenase levels as a prognostic factor was confirmed by the multivariate analysis in 19 of the included studies [16, 17, 19–23, 25, 27, 28, 30, 32, 34, 35, 38, 40–43]. An HR for overall survival (OS) and progression-free survival (PFS) was extracted from 27 and 8 studies, respectively. Funnel plot analyses did not reveal a significant publication bias regarding the analyzed outcomes (Additional file 3: Figure S1). However, the funnel plot B (PFS) does not allow to exclude a publication bias, because of limited number of studies.

### The prognostic value of lactate dehydrogenase levels

**Fig. 2:**
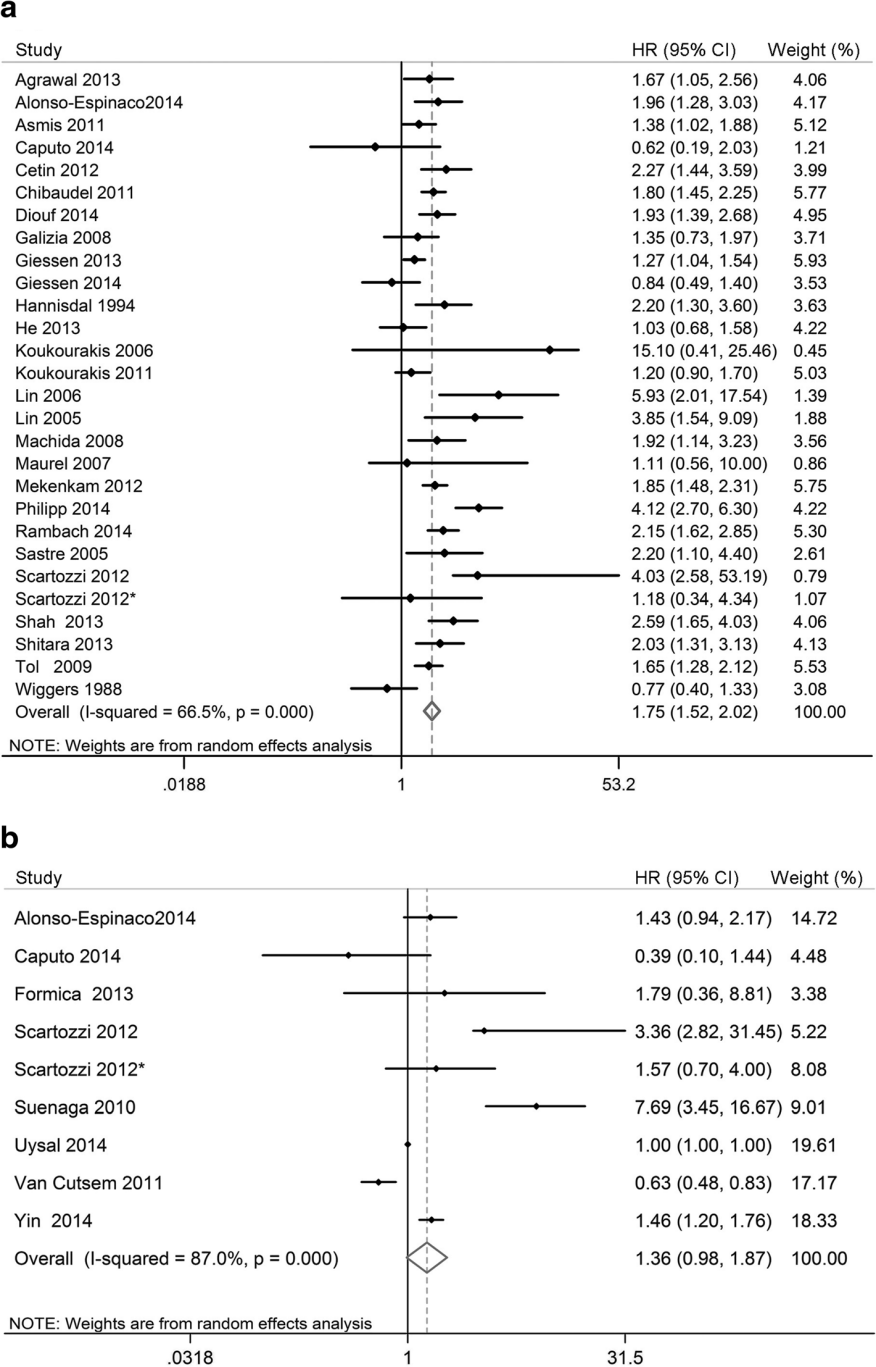
Meta-analyses of the association between lactate dehydrogenase levels and (**a**) overall survival or (**b**) progression-free survival. Squares and horizontal bars indicate the point estimates (HRs) with 95 % CIs for each individual study. Diamonds indicate the summary estimates for the hazard ratio. The width of the diamond corresponds to the 95 % CI

Pooled analysis of OS in all studies using the random effects model revealed a significant prognostic value for lactate dehydrogenase levels in CRC patients (HR, 1.75; 95 % CI, 1.52–2.02; *n* = 27; I^2^ = 66.5 %; Fig. 2a). Sensitivity analyses revealed that heterogeneity was not caused by any one study. However, our meta-analyses using the random effects model did not confirm the prognostic value for lactate dehydrogenase levels in predicting PFS (HR, 1.36; 95 % CI, 0.98–1.87; *n* = 8; I^2^ = 87 %; Fig. 2b), and we observed a significant degree of heterogeneity. This heterogeneity could not be reduced substantially by the exclusion of any one study.

### Subgroup analyses

Despite the limited number of included studies, the subgroup analyses of lactate dehydrogenase levels and survival were performed to thoroughly explore the results. We performed meta-regression and subgroup analysis of lactate dehydrogenase levels on OS according to study location, patient age, number of patients, metastasis, chemotherapy with anti-angiogenesis drugs, study type, and risk of bias. The results revealed that none of the investigated factors had a significant association with the heterogeneity (Table 2). However, subgroup analysis indicated a significant relation between high lactate dehydrogenase levels and reduced OS among metastatic CRC patients (HR, 1.96; 95 % CI, 1.61–2.37), although this effect was not significant among non-metastatic patients (HR, 1.21; 95 % CI, 0.79–1.86; Table 2). The effect of LDH on OS among different cutoffs for LDH is also shown in Table 2. The HRs were 1.93 (95 % CI 1.50 to 2.49) for LDH cutoff >300U/L, 1.84(95 % CI 1.08 to 3.13) for LDH cutoff 250 to 300U/L and 1.44 (95 % CI 0.94 to 2.21) for LDH cutoff <250U/L. There was no statistically significant heterogeneity between the different cutoffs for LDH (P for subgroup difference = 0.309). Our results suggest that relation between high lactate dehydrogenase levels and reduced OS among metastatic CRC patients disappears if the LDH cutoff value less than 250U/L (HR, 1.44; 95 % CI 0.94 to 2.21).

**Table 2:** Stratified analysis of pooled hazard ratios of lactate dehydrogenase on overall survival

			Pooled HR (95 % CI)			Heterogeneity	
Stratified analysis	No. of studies	No. of patients	Fixed	Random	Meta-regression on *p*-value	I^2^ (%)	*p*-value
Study location					0.581		
Asia	4	580	1.66 [1.29, 2.14]	1.82 [1.14, 2.9]		67.9	0.025
Europe	19	5276	1.66 [1.53, 1.80]	1.67 [1.40, 2.0]		69.5	<0.001
Other regions	5	1065	1.85 [1.52, 2.25]	2.07 [1.45, 2.94]		64.1	0.025
Age					0.563		
≤ 50	2	191	1.98 [1.33, 2.94]	2.31 [1.04, 5.13]		63.1	0.1
No limitation	22	5623	1.70 [1.57, 1.84]	1.77 [1.51, 2.08]		68.5	<0.001
Number of patients					0.68		
≥ 100	22	6428	1.68 [1.56, 1.81]	1.73 [1.49, 2.01]		69	<0.001
< 100	6	439	1.84 [1.66, 2.04]	1.96 [1.11, 3.43]		60.3	0.28
Metastasis					0.059		
Yes	16	5044	1.84 [1.66, 2.04]	1.96 [1.61, 2.37]		64.4	<0.001
No	5	883	1.53 [1.29, 1.82]	1.21 [0.79, 1.86]		74.4	0.028
LDH cutoff					0.309		
> 300 U/L	7	764	1.93 [1.50, 2.49]	1.98 [1.41, 2.77]		29.1	0.206
250–300 U/L	5	1028	1.61 [1.38, 1.88]	1.84 [1.08, 3.13]		88.6	<0.001
< 250 U/L	6	1174	1.58 [1.31, 1.90]	1.44 [0.94, 2.21]		75.4	0.001
Chemotherapy with anti-angiogenesis drugs					0.64		
Yes	5	1675	1.75 [1.51, 2.02]	1.78 [1.41, 2.23]		57.3	0.053
No	16	4166	1.60 [1.46, 1.75]	1.65 [1.40, 1.94]		54.8	0.003
Study type					0.863		
non-RCT^a^	22	3683	1.66 [1.51, 2.02]	2.03 [1.31, 3.13]		71.5	<0.001
RCT	5	3238	1.73 [1.54, 1.94]	1.73 [1.54, 1.94]		<0.01	0.535
Risk of bias					0.31		
High	16	3142	1.52 [1.36, 1.68]	1.63 [1.28, 2.09]		76.5	<0.001
Low	11	3799	1.87 [1.69, 2.07]	1.65 [1.28, 2.12]		<0.01	0.655

^a^non-RCT includes PSCS, RMCS and RSCS groups

Subgroup analysis of the other factors did not alter the significant prognostic value of lactate dehydrogenase levels in predicting OS.

We also performed meta-regression and subgroup analysis of lactate dehydrogenase levels and PFS. Owing to the limited number of included studies, only study location, number of patients, chemotherapy with anti-angiogenesis drugs, and risk of bias were explored. The results revealed that none of the investigated factors had a significant association with the heterogeneity (Table 3). Moreover, subgroup analysis revealed no relationship between lactate dehydrogenase levels and PFS among CRC patients.

**Table 3:** Stratified analysis of pooled harazd ratios of lactate dehydrogenase on progression free survival

			Pooled HR (95 % CI)			Heterogeneity	
Stratified analysis	No. of studies	No. of patients	Fixed	Random	Meta-regression on *p*-value	I^2^ (%)	*p*-value
Study location					0.196		
Asia	2	418	1.60 [1.33, 1.93]	3.20 [0.63,16.27]		93.8	<0.001
Europe	6	1359	0.87 [0.71, 1.08]	1.15 [0.65, 2.04]		74.4	0.002
Number of patients					0.762		
≥100	4	1483	1.16 [1.00, 1.34]	1.26 [0.72, 2.19]		89.5	<0.001
<100	5	330	1.00 [1.001, 1.004]	1.59 [0.64, 3.98]		86.3	<0.002
Chemotherapy with anti-angiogenesis drugs					0.717		
Yes	6	1422	1.00 [1.001, 1.004]	1.36 [0.96, 1.98]		90.6	<0.001
No	2	295	1.56 [1.06, 2.33]	1.80 [0.86, 3.80]		41.9	0.19
Risk of bias					0.805		
High	6	738	1.00 [1.001, 1.004]	1.51 [1.01, 2.25]		89.1	<0.001
Low	3	1075	0.74 [0.57, 0.95]	1.31 [0.49, 3.53]		805	0.006

## Discussion

This systematic review and meta-analysis revealed that high lactate dehydrogenase levels are associated with poor OS among patients with CRC. However, this prognostic value was not observed for PFS among CRC patients.

Despite the number of studies that have been conducted in this field, the prognostic value of lactate dehydrogenase levels among CRC patients has remained highly uncertain, given the inconsistent results from the previous studies. In the present study, pooled analyses of the available data revealed a significant association between high lactate dehydrogenase levels and poorer OS. However, there was insufficient statistical power to detect this association among patients with non-metastatic disease (Pooled HR1.21, 95 % CI [0.79, 1.86]).

There is recent evidence that the addition of anti-angiogenesis medication diminishes the impact of lactate dehydrogenase expression on the prognosis of CRC patients [30]. Besides, recent research reveals that high LDH is a significant indicator of bevacizumab-based chemotherapy-induced response to treatment for previously untreated metastatic colorectal cancer patients [60]. However, our meta-analysis did not detect a similar effect among CRC patients. This discrepancy may be attributed to the different kinds of anti-angiogenesis medications that were used in the previous study. Combined with the different dose that was employed for the anti-angiogenesis medications, there was insufficient statistical power to detect any differences in the survival of CRC patients (*p* = 0.64). However, our data supports the approach to aggregate results from the available studies regarding the prognostic significance of anti-angiogenesis drugs in CRC.

Interestingly, we detected significant heterogeneity among the studies that were included in this systematic review. However, sensitivity analysis did not identify the source of this heterogeneity. We did observe a wide range in the cut-off levels for lactate dehydrogenase; therefore, additional standardization should be addressed in the design of future studies, thereby enhancing the utility of their results. Most of the studies that we included focused on metastatic CRC patients, which could also be a source of bias. In addition, our approach of extrapolating the HRs from the survival plots might be another potential source of bias. Although we extracted the survival rates from survival curve graphs using Engauge software, this approach did not completely eliminate inaccuracies during the extraction of the survival rates. Moreover, the language of publication may have added additional bias, as the present review was restricted to articles published in English, German, or French, as other languages were not accessible for the readers. This bias could favor positive studies, which are more frequently published in English, as negative studies tend to be published in the authors’ native languages.

## Conclusions

In conclusion, there is evidence that high lactate dehydrogenase levels indicate poor prognosis among CRC patients. However, subgroup analysis revealed no such prognostic value among non-metastatic CRC patients. These findings should encourage efforts to identify subpopulations with high lactate dehydrogenase levels that might put metastatic patients at a particular risk of poor survival.

### Availability of data and materials

The datasets supporting the conclusions of this article are included within the article and its additional files.

## Additional files

Additional file 1: Search strategies. (DOCX 14 kb) Additional file 2: Assessment of risk of bias. (XLSX 11 kb) Additional file 3: Figure S1. Funnel plot analyses of studies report OS (A) and PFS (B). (JPEG 1290 kb)

## Abbreviations

CRC: Colorectal cancer
OS: Overall survival
PFS: progression free survival
