# Supplementary material for: The prognostic value of lactate dehydrogenase levels in colorectal cancer: a meta-analysis
Source: BMC Cancer. 2016 Mar 25;16:249. doi: 10.1186/s12885-016-2276-3 (PMC4807548; doi:10.1186/s12885-016-2276-3)
Supplement: Additional file 1: — Search strategies. (DOCX 14 kb) [file 12885_2016_2276_MOESM1_ESM.docx]

**The prognostic value of lactate dehydrogenase levels in colorectal cancer: A meta-analysis**

Search strategies

Embase No date limited

Search Date 2014-09-21

| **#** | **Searches** | **Results** |
| --- | --- | --- |
| 1 | exp Colorectal Neoplasms/ | 148,719 |
|  | (((cancer* or carcinom* or neoplas* or tumo* or adeno*[Title]))) AND ((rectal or rectum or colon* or colorect* or colo-rect*[Title])) | 124,470 |
| 2 |  |  |
| 3 | 1 or 2 | 196,019 |
| 4 | exp Lactate dehydrogenase/ | 78,838 |
| 5 | LDH OR LDH* | 2,088 |
| 6 | 4 or 5 | 79,017 |
| 7 | 3 and 6 | 603 |
| 8 | mortality'/exp | 671,502 |
| 9 | survival'/exp | 626,181 |
| 10 | prognosis'/exp | 458,131 |
| 11 | prognos*:ti OR risk:ti OR survival:ti OR recurren*:ti OR mortality:ti OR predict*:ti OR outcome*:ti OR significan*:ti OR indicator*:ti OR impact:ti OR detect*:ti OR relevan*:ti |  |
|  |  | 1,814,242 |
| 12 | or/8-11 | 2,791,295 |
| 14 | 7 and 12 | 270 |
| 15 | #14 [animals]/lim | 10 |
| 16 | 14 not 15 | 260 |
| 17 | remove duplicates from 16 | 260 |

The detailed search strategies applied in the further databases are available upon request.
