# Supplementary figures and images for: The prognostic value of lactate dehydrogenase levels in colorectal cancer: a meta-analysis
Source: BMC Cancer. 2016 Mar 25;16:249. doi: 10.1186/s12885-016-2276-3 (PMC4807548; doi:10.1186/s12885-016-2276-3)

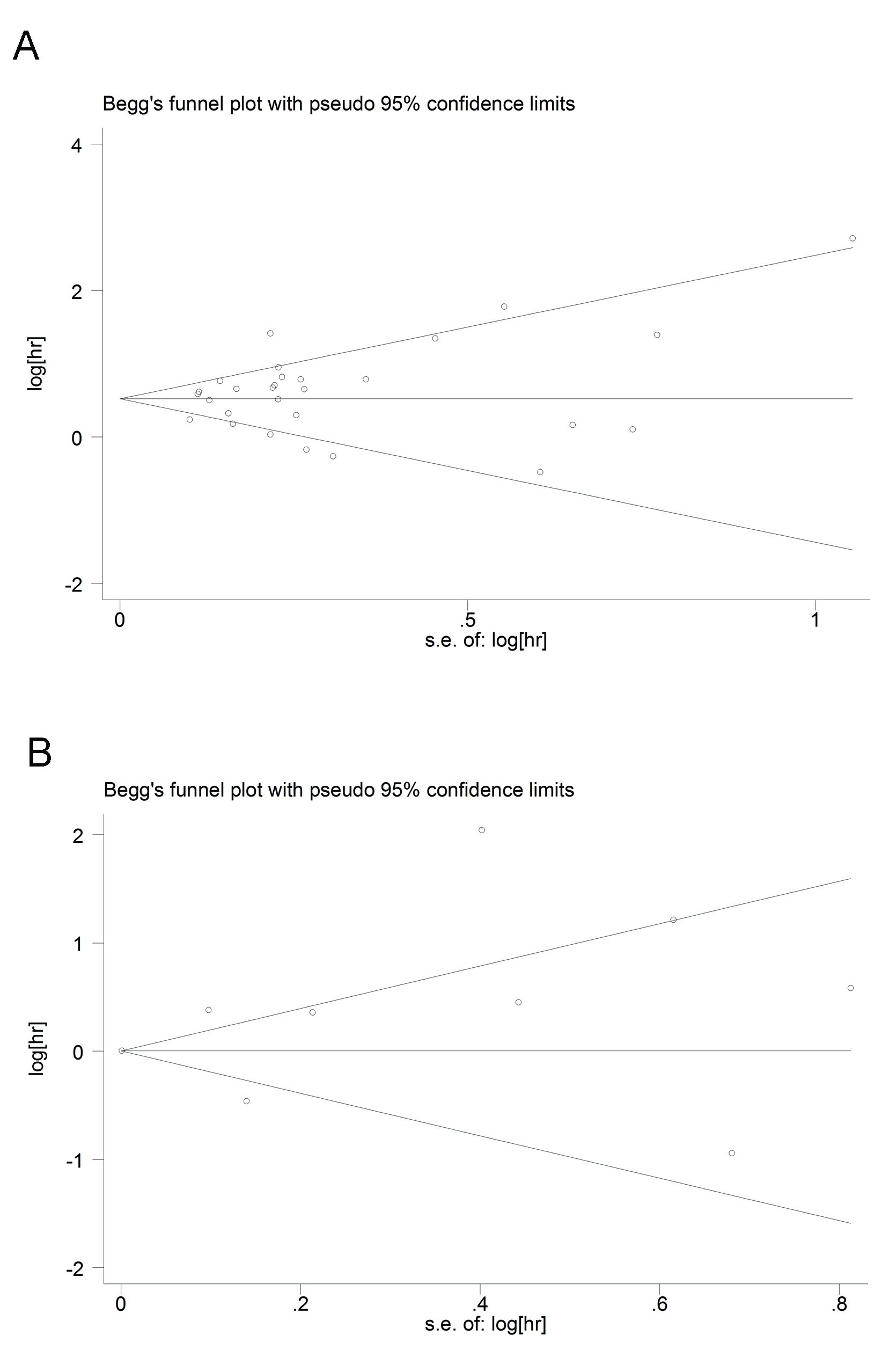

Supplement: Additional file 3: Figure S1. — Funnel plot analyses of studies report OS (A) and PFS (B). (JPEG 1290 kb) [file 12885_2016_2276_MOESM3_ESM.jpeg]
